# Frequency of Lost Dogs and Cats in the United States and the Methods Used to Locate Them

**DOI:** 10.3390/ani2020301

**Published:** 2012-06-13

**Authors:** Emily Weiss, Margaret Slater, Linda Lord

**Affiliations:** 1 Shelter Research and Development, Community Outreach, American Society for the Prevention of Cruelty to Animals (ASPCA^®^), 6260 N. Hillside, Wichita, KS 67219, USA; E-Mail: emily.weiss@aspca.org; 2 Shelter Research and Development, Community Outreach, American Society for the Prevention of Cruelty to Animals (ASPCA^®^), 50 Stone Ridge Drive, Northampton, MA 01602, USA; 3 Department of Veterinary Preventive Medicine, College of Veterinary Medicine, The Ohio State University, Columbus, OH 43210, USA; E-Mail: linda.lord@cvm.osu.edu

**Keywords:** lost dog, lost cat, collar, identification tag, stray cat, stray dog

## Abstract

**Simple Summary:**

Dogs and cats are a common member of the family in homes across the US. No population-based data exist on the frequency of pets getting lost from the home and lost pets can be a source of human and animal suffering. Our primary objective was to determine the percentage of owned dogs and cats that were lost, and of these, what percentages of pets were recovered. We examined the recovery success for dogs compared to cats and the methods used as well as the relationship between lost or found pets and pet and owner demographics. While 15% of dog and cat owners lost their pets, dogs had higher recovery rates (93%) than cats (75%) as well as being returned using different search methods.

**Abstract:**

A cross-sectional national random digit dial telephone interview was conducted between September and November 2010. There were 1,015 households that had owned a dog or cat within the past five years. Of these 817 households owned dogs and 506 owned cats. Fourteen percent of dogs (95% Confidence Interval (CI): 11–16%) and 15% (95% CI: 12–18%) of cats were lost in the past five years. No owner demographic variables were associated with losing a pet. Ninety three percent (95% CI: 86–97%) of dogs and 75% (95% CI: 64–85%) of cats were recovered. For dogs, searching the neighborhood and returning on their own were the most common methods of finding the dog; 14% were found through an identification tag. For cats, returning on their own was most common. Dogs were more likely than cats to be lost more than once. Cats were less likely than dogs to have any type of identification. Knowledge of the successful methods of finding dogs and cats can provide invaluable help for owners of lost pets. Since 25% of lost cats were not found, other methods of reuniting cats and their owners are needed. Collars and ID tags or humane trapping could be valuable approaches.

## 1. Introduction

Dogs and cats continue to be a common component of the family in homes across the United States as well as in many other countries with 86.4 million cats and 78.2 million dogs owned in the US [1]. Given the numbers of pets and frequent anecdotes from pet professionals regarding lost pets, pets are lost from their homes regularly. How often this occurs and how many pets are reunited with their owners is still unclear. Many dogs and cats likely return home on their own or are found and returned home by neighbors. Others may enter the animal sheltering system. Previous research shows that many members of the community view finding the owners of lost pets as an important goal, and one well worth the effort [2]. However, among people who found a pet and attempted to find the owner by contacting an animal shelter or placing an advertisement in the local newspaper, only 38% of these finders were able to reunite pets with their owners, with dogs much more likely to be returned to their owners (46%) than cats (3%) [2]. 

In many US shelters, more than half of the total intake may be stray animals, typically defined as a pet not relinquished to a shelter by the owner or brought in as part of a legal seizure [3]. With the number of dogs and cats entering shelters per year in the US estimated to be between 5 and 7 million, the time and funding spent to reunite presumed lost pets with their owners can be quite significant [3]. Data suggests that the frequency of lost pets reclaimed from shelters by owners in most communities hovers between 10–30% for dogs and less than 5% for cats in the US [4] and 40% dogs in Australia [5]. This means that the majority of stray dogs and cats are never reunited with an owner once they enter an animal shelter. 

Two publications identified participants by searching newspaper advertisements and contacting animal control facilities to study the reunion of dogs and cats with their owners [6,7]. These studies therefore excluded owners who did not choose these methods to find their pets. These studies demonstrated that the recovery of lost dogs and cats differed, with dog owners more likely to find their companions at an animal welfare organization, and cat owners more likely to have their pets return home on their own or to have found them in the neighborhood despite contacting animal control agencies. 

To our knowledge, there are no national or international studies investigating lost pets. A national study focusing on the frequency that owners loose their pets as well as a preliminary description of the experiences of those pet owners could help to drive additional programs and practice regarding search methods. The primary objectives of the current project were to calculate a population-based estimate of the percentage of owned dogs and cats that were lost from the home in a five year period. The secondary objectives were to estimate the percentage of lost pets subsequently recovered by their owners for dogs *vs.* cats and the methods used as well as examine basic pet and respondent demographic variables for their associations with lost and found pets. 

## 2. Methods

### 2.1. Survey Administration

A professional survey research company (Strategic Research Group, Columbus, OH, USA) was hired to conduct a national random digit dialing (RDD) telephone survey using land-lines to identify households who had owned and lost a dog or cat in the past five years. Five years was chosen as the interval as it was a reasonable time frame for recent memory of a lost pet. Only English-speaking respondents in homes with land-lines were eligible. If answering machines were reached, a message was left to call and interviewers were available from 9 AM until 9:30 PM EST. The telephone survey was conducted from September through November 2010. The national RDD sample was supplied by Genesys Survey Sampling (Marketing Systems Group, Fort Washington, PA, USA). The study was considered exempt from human subjects review by University of Illinois Institutional Review Board guidelines.

### 2.2. Questionnaire

The initial questionnaire was developed by the authors and reviewed by a number of individuals familiar with the field of animal sheltering. An initial pilot study of 25 interviews was conducted and final revisions to the survey were made. The goal for the total number of lost pets was 400 in order to obtain a 95% confidence interval of not more than ±5% for the estimates of percentage of dogs or cats lost assuming 50:50 lost dogs: lost cats. Based on a 50% pet ownership rate nationally and our estimate of pets being lost of 30%, a household sample size of 2,666 calculated and the budget established accordingly using standard statistical software (Stata 11.1, StataCorp LP, College Station, TX, USA). 

The questionnaire consisted of initial screening questions to identify qualified participants for the survey. The interviewer first asked the household respondent whether the household had owned a dog or cat in the past five years. If the household had not owned a dog or cat within the last five years, the survey was terminated. If the household had owned a dog or cat, the interviewer asked to speak with someone with knowledge about the pets in the household. This respondent was then asked about the number of dogs and cats owned and one dog/cat was selected to participate (the dog and/or cat whose name began with letter closer to “A” in the alphabet was chosen). Additional questions about the chosen pet were the pet’s sex, if the chosen pet was spayed or neutered and a question to determine if that pet had ever been lost in the past five years. This included pets no longer in the household. The exact wording for the question about whether a pet was lost was: “The next series of questions have to do with whether your pet has ever become lost. Examples of this include: your pet ran away from home, your pet was gone longer than expected, your pet didn’t come home when he/she usually does, your pet escaped from the yard or house.” If no dog/cat was lost, the survey was terminated at this point. 

Households could have up to one lost dog and one lost cat included in the survey. If a dog/cat was lost, the interviewer asked a series of questions about the chosen dog/cat. Questions included the number of times lost, and for the most recent time the pet was lost, the types of identification (ID) worn and/or implanted, if the pet was found, if so, how and if the pet was not found what search methods were used by the owner. Additional demographic questions were also asked for respondent’s education level, income level and gender. The interview was conducted with an adult 18 years or older knowledgeable about the pet.

### 2.3. Data Collection

All telephone interviews were conducted by the survey research company’s trained personnel who used a standard computer-assisted telephone interviewing software program (CATI Software). All interviewers were regularly monitored by supervisors. During the calling phase, at least six attempts at different times of the day and on different days were made before the participant was classified as a non-responder. 

### 2.4. Statistical Analysis

Response rate was calculated using the American Association of Public Opinion Research guidelines [8]. Proportions were calculated for responses that consisted of categorical data. All percentages had 95% exact binomial confidence intervals calculated and provided in parentheses following the point estimate. For comparisons of dog *vs*. cat responses, the chi-square test was used, except that the Fisher exact test was used when the expected value for any cell was <5. The number of dog and cat owning households by state from a national representative panel were ranked and averaged [9]. That ranking was compared to the ranking of dog and cat households by state in this study using the Wilcoxon signed rank test. 

Univariable logistic regression was used to identify factors potentially associated with whether a dog or cat had ever been lost; separate analyses were performed for responses for dogs and cats. Demographic variables evaluated were the pet’s sex and neuter status and the owner’s education, income and gender. Education was collapsed from nine categories into four. Income was collapsed from ten categories to four (Table 2). All variables with *P* ≤ 0.25 in univariable analyses were subsequently included in multivariable logistic regression analyses. Variables with the greatest *P*-values were sequentially removed from the multivariable model using backwards, stepwise manual elimination until the p-values of all remaining variables were *P* ≤ 0.05. Meaningful interactions between the main effect variables in the model (those that could have a plausible explanation) were tested for inclusion in a similar manner. The goodness of fit of the final model was tested with the Hosmer-Lemeshow goodness-of-fit test. For all analyses including Hosmer-Lemeshow, values of *P* ≤ 0.05 were considered significant. Standard statistical software was used (Stata 11.1, StataCorp LP, College Station, TX, USA).

For comparisons of demographic variables (pet’s sex and neuter status and the owner’s education, income and gender) and whether or not the lost pet was reunited with the owner, the chi-square test was used, except that the Fisher exact test was used when the expected value for any cell was <5 with cats and dogs analyzed separately. Owner education and income were collapsed as above. Relationships between pet demographic variables and human demographic variables were also tested. Due to the small sample size, no additional multivariable analyses were attempted. *P* ≤ 0.05 was considered statistically significant for all analyses. 

## 3. Results

There were 6,996 calls made of which only 4,029 were households. There were 434 (11%) messages left on the answering machine with no person reached, 331 (8%) refusals to participate, 326 (8%) households contacted but no interview performed for various reasons, 257 (7%) households where eligibility could not be determined and 94 (2%) where a language other than English was spoken. There were 2,587 households successfully contacted between 13 September 2010 and 23 November 2010. The response rate was 64% (2,587/4,029) among households called. While our original target was 2,666, that target was based on an estimate of the pet owners we would be able to reach in comparison to the total calls we made. The actual percentage was lower than our estimate. Of these households, 1,015 (39%, 37–41%) had owned a dog or cat in the past five years. Sixty five percent of respondents (62–68%) were female. Eight hundred and seventeen households owned dogs and 506 owned cats, among them 308 owned both dogs and cats. One respondent did not provide information beyond owning a pet, for a total of 1,014 households with pet data. The median number of owned dogs during this time was one (range, one to 22). The median number of owned cats was two (range, one to 27). Fifty one percent of dogs and 48% of cats were male. Eighty percent of dogs (77–83%) and 88% of cats (85–91%) were spayed or neutered (*P* < 0.001 for the proportion of dogs *vs*. cats). Male dogs were significantly less likely to be sterilized than female dogs (*P* = 0.002) while there was no significant difference between the percentage of male and female cats that were sterilized. Data about the frequency of pets getting lost, type of ID worn during the most recent episode and the methods used to find pets are in Table 1. Of all households, 15% (13–18%) had lost a dog and/or cat and of those, 85% (79–90%) recovered the pet. Cats were less likely to be recovered than dogs and there were some significantly different methods used both for successful and unsuccessful recoveries. Of the 18 cats who were never found, 15 (83%, 59–96%) had no personalized ID and of those, 12 cats (66%, 41–87%) had no license or rabies tag. Dog or cat owners participated from all states except Alaska, Delaware, Hawaii or Wyoming. The five states in which most respondents were located were California, Texas, New York, Florida and Pennsylvania. Twelve percent of respondents did not provide usable zip codes to identify their states. When the frequency of our respondents’ states of residency were ranked, the rankings were found to be similar to the national ranking (*P* = 0.7) [9]. Median income in this study was $50,000 to $74,999 but 31% of respondents did not provide an answer to this question. 

**Table 1:** A national survey of 1,014 households owning dogs or cats in the past five years ^a^.

Question	Number of Dogs	Percentage of Dogs	Number of Cats	Percentage of Cats	P-Value ^b^ for dogs compared to cats
Has your pet ever become lost in the past five years?					0.6
Yes	110	14 (11–16)	74	15 (12–18)	
No	705	87 (84–89)	429	85 (82–88)	
Total	815 ^c^	100	503	100	
Refused	1		2		
Don’t know	0		1		
If yes, how many times was the pet lost?					0.05
1 time	52	49 (39–58)	51	69 (57–79)	
2–5 times	38	36 (27–45)	15	20 (12–31)	
6–10 times	12	11 (6–19)	5	7 (3–15)	
More than 10 times	5	6 (2–11)	3	4 (1–11)	
Total	107	100	74	100	
Don’t know	3				
					
Which of the following was your pet wearing the last time he/she was lost? (more than one could be worn) n = dog sample size; cat sample size					
Wearing a collar (n = 109; 72)	98	90 (83–95)	31	43 (31–55)	<0.001
Wearing a rabies tag (n = 106; 70)	74	70 (50–78)	19	27 (17–39)	<0.001
Wearing a license (n = 104; 72)	60	58 (48–67)	15	21 (12–32)	<0.001
Wearing a personalized ID tag with phone number (n = 109; 72)	67	61 (52–71)	18	25 (16–37)	<0.001
Had a microchip (n = 103; 72)	25	24 (16–34)	11	15 (8–26)	0.2
No rabies, license or ID tag or microchip (n = 101; 71)	11	11 (6–19)	40	56 (44–68)	<0.001
					
Did you find your pet?					0.002
Yes	101	93 (86–97)	55	75 (65–85)	
No	8	7 (3–14)	18	25 (15–36)	
Total	109	100	73	100	
Don’t know	1		0		
Refused	0		1		
What was the primary method used to find the pet (when successful)?					<0.001
I found my pet by searching my neighborhood	49	49 (38–59)	16	30 (18–44)	
My pet returned on its own	20	20 (13–29)	32	59 (45–72)	
I was contacted because of a tag my pet was wearing/my pet’s microchip	15	15 (9–23)	1	2 (0.04–10)	
Neighbor brought pet home	7	7 (3–14)	0	0	
I found my pet by visiting/contacting animal control	6	6 (2–12)	1	2 (0.04–10)	
Other	4	4 (1–10)	4	7 (2–18)	
Total	101	100	54	100	
Refused			1		
					
What methods were used to attempt to find pet (unsuccessful)? More than one answer was possible					
Waited for pet to come home	6	75 (35–97)	14	78 (52–94)	1.0
Searched neighborhood	6	75 (35–97)	12	67 (41–87)	1.0
Visited shelter	6	75 (35–97)	4	22 (6–48)	0.03
Hung posters	4	50 (16–84)	3	17 (4–41)	0.2
Ad in paper	4	50 (16–84)	2	11 (1–35)	0.06
Posted online	4	50 (16–84)	1	6 (1–27)	0.02
Called veterinary or other professionals	3	38	2	11	0.3
Other	0	0	1	6	–
Refused	0		1		

^a ^Up to one dog and one cat per household were eligible. ^b ^Chi-square test or Fisher exact test *P*-value. ^c ^One household that owned dogs did not provide any additional details on the dogs.

**Table 2:** Pet and respondent demographic variables analyzed by whether they lost or their pets or not ^a^.

Question	Dog owners who lost dogs (n = 110)	Percentage and 95% confidence interval	Dog owners who did not lose dogs (n = 705)	Percentage and 95% confidence interval	Cat owners who lost cats (n = 74)	Percentage and 95% confidence interval	Cat owners who did not lose cats (n = 429)	Percentage and 95% confidence interval
**Pet Demographics**								
*Sex*				0.5 ^b^				0.7
Male	59	54 (44–63)	354	50 (47–54)	33	46 (34–58)	206	48 (43–53)
Female	51	46 (39–56)	349	50 (46–53)	39	54 (42–66)	221	52 (47–56)
Don’t know	0		2		2		1	
Refused	0		0		0		1	
								
*Neuter status*				0.4				0.7
Yes	91	83 (74–89)	555	80 (76–83)	65	90 (81–96)	369	88 (84–91)
No	19	17 (11–26)	142	20 (17–24)	7	10 (4–19)	52	12 (9–16)
Don’t know	0		4		2		6	
Refused	0		4		0		2	
								
**Owner Demographics**								
*Education Level *				0.6				0.8
High school graduate or less	30	28 (20–37)	202	32 (29–36)	21	29 (19–41)	117	30 (26–35)
Some college or trade/tech/vocational	27	25 (17–34)	140	22 (19–26)	21	29 (19–41)	59	15 (12–19)
College graduate	36	33 (25–43)	191	30 (27–34)	18	25 (16–37)	115	30 (25–35)
Any graduate work	15	14 (8–22)	94	15 (12–18)	12	17 (9–27)	63	16 (13–20)
Don’t know	0		6		0		2	
Refused	2		72		2		43	
*Income *			0.3				0.7	
Less than $30,000	35	41 (30–52)	141	29 (25–34)	19	31 (20–44)	105	36 (31–42)
$30,000 to $49,999	15	17 (10–27)	122	25 (22–30)	16	26 (16–39)	71	24 (20–30)
$50,000 to $99,999	22	26 (17–36)	144	30 (26–34)	19	31 (20–44)	71	24 (20–30)
$100,000 and over	14	16 (9–26)	72	15 (12–19)	7	11 (5–22)	43	15 (11–19)
Don’t know	4		42		5		26	
Refused	20		184		8		113	
								
*Gender*			0.12				0.17	
Men	47	43 (33–52)	247	35 (32–39)	19	26 (16–37)	144	34 (29–38)
Women	63	57 (47–67)	458	65 (61–68)	55	74 (63–84)	285	66 (62–71)

^a^ From interviews with 1,015 dog and cat owners in the past five years. ^b^ Univariable logistic regression *P*-values are opposite the variable names.

**Table 3:** Demographic variables for households with lost pets analyzed by whether or not pets were recovered ^a^.

Question	Dog owners who found their lost dogs (n = 101)	Percentage and 95% confidence interval	Dog owners who did not find their lost dogs (n = 8)	Percentage and 95% confidence interval	Cat owners who found their lost cats (n = 55)	Percentage and 95% confidence interval	Cat owners who did find their lost cats (n = 18)	Percentage and 95% confidence interval
**Pet Demographics**								
*Sex*				1.0 ^b^				0.6
Male	55	54 (44–63)	4	50 (47–54)	27	49 (35–63)	6	38 (15–65)
Female	46	46 (39–56)	4	50 (46–53)	28	51 (39–65)	10	62 (35–85)
Don’t know	0		0		0		2	
Refused	0		0		0		0	
*Neuter status*				0.03				0.007
Yes	86	85 (77–91)	4	50 (10–56)	52	96 (87–100)	12	71 (44–90)
No	15	15 (9–23)	4	50 (10–56)	2	4 (0.4–13)	5	29 (10–56)
Don’t know	0		0		1		1	
Refused	0		0		0		0	
								
**Owner Demographics**								
*Education Level *				0.03				0.03
High school graduate or less	24	24 (16–34)	6	75 (35–97)	12	23 (13–37)	9	50 (26–74)
Some college or trade/tech/vocational	26	26 (18–36)	1	13 (3–53)	15	29 (17–43)	6	33 (13–59)
College graduate	34	35 (25–45)	1	13 (3–53)	15	29 (17–43)	3	17 (4–41)
Any graduate work	15	14 (9–24)	0	0 (0–37)	12	23 (13–37)	0	0 (0–19)
Don’t know	1		0		2		0	
Refused	2		0		1		0	
								
*Income *				0.15				0.001
Less than $30,000	28	35 (25–47)	7	100 (59–100)	8	18 (8–32)	11	69 (41–89)
$30,000 to $49,999	15	19 (11–29)	0	0 (0–41)	12	27 (15–42)	4	25 (7–52)
$50,000 to $99,999	22	28 (18–39)	0	0 (0–41)	18	40 (26–56)	1	6 (0.2–30)
$100,000 and over	14	18 (10–28)	0	0 (0–41)	7	16 (6–29)	0	0 (0–21)
Don’t know	3		1		4		1	
Refused	19		0		6		1	
								
*Sex*				0.7				0.7
Men	43	43 (33–52)	4	35 (32–39)	15	26 (16–37)	4	34 (29-38)
Women	58	57 (47–67)	4	65 (61–68)	40	74 (63–84)	14	66 (62-71)

^a^ Data include cat (73) and dog (109) owners in the past five years whose dogs or cats were lost. ^b^ Fisher exact test *P*-values are opposite the variable names.

There were no significant variables in the multivariable logistic regression models examining whether the pet was lost or not and the demographic variables (Table 2). However, there were significant associations between finding the pet or not and pet neuter status, owner education (both dogs and cats), and income (for cats) (Table 3). When examining the association between pet and owner demographic variables and whether or not the owner had lost a pet, there were no significant associations between the cat’s sex and human demographic variables for the full data set (owner education *P* = 0.9; income *P* = 0.2; gender of owner *P* = 0.1) or for the dog’s sex (owner education *P* = 0.9; income *P* = 0.6; gender of owner *P* = 0.7). However, neuter status of cats was significantly associated with owner education (*P* = 0.004) and income (*P* = 0.006) but not owner gender (*P* = 0.9). Dog neuter status was similarly associated with owner education (*P* < 0.001) and income (*P* < 0.001) but not owner gender (*P* = 0.1). In all cases, higher education or income respondents were more likely to have their cats or dogs neutered and lower education or income respondents were more likely to have intact pets.

## 4. Discussion

The definition of lost pets used in this study deliberately was designed to be broad so that owners of pets would include any time they were concerned about the absence of the pet from the home. This does mean that the responses for a lost pet were based on the owner’s concern and belief that the pet was lost and not an objective definition. Likely, a number of pets whose owners believed them lost would not have been considered lost by other owners. No time period was included because the normal activities of some pets would include being out roaming loose for variable periods of time. All owners considered the pets reported here to be lost in that they were gone when they would not normally be absent from the home or yard. We do not know what length of time was used by each owner to determine whether the pet was lost. This likely varies by species and whether the pet was normally allowed to roam freely or not. We sought a comprehensive definition that would apply to different situations depending on how pets were normally kept. 

Both dogs and cats were reported as being lost at a similar percentage, 14% (11–16%) and 15% (12–18), respectively. Of the animals lost, the majority were recovered, with dogs reunited with their owners 93% (86–97%) of the time and cats, 75% (63–84%). Dogs were more likely than cats to be lost more than once. Cats were less likely than dogs to have any type of ID or even a collar. The relatively high prevalence of ID overall in this study may be due to the question referring to the most recent time the pet became lost; it is plausible that previous experience with a lost pet might encourage owners to provide ID. However, this could also be due to owners whose pets have ID being more concerned about their pets getting lost or considering their pets lost sooner.

We did not ask pet owners if their cats were indoor/outdoor or if their dogs were allowed to roam the neighborhood or were in backyards. These factors may influence the likelihood a pet was lost, or perceived as lost. The specific circumstances under which the pet was considered to be lost would also be useful. This is an area of future research that should be explored. 

Unfortunately, both our estimated pet ownership percentage and estimate of lost dogs and cats within the past 5 years were lower than expected (39% *vs*. 50% and 15% *vs*. 30%). This resulted in fewer than 200 lost pets included in the survey. This seriously limited our ability to analyze less common responses including the role of identification tags in getting lost pets reunited with their owners. 

Our logistic regression analysis did not find that basic pet or owner demographics were associated with pets getting lost. To have found a statistically significant difference for owner gender and lost cats, a total sample size for cat owners of 2,400 (80% power, alpha = 0.05) would have been needed. While we did not have a large enough sample size for adequate power, the small differences for the demographic variables between pets that were lost and those that were not for both dogs and cats make the demographic variables examined less likely to be of practical importance in predicting which pets get lost. This implies that more complex variables relating to pet environments and owner lifestyles will need to be studied to better understand why pets get lost. 

The interviewer asked to speak with a respondent who was knowledgeable about the pets in the household. This was the person who then provided the owner demographic data. While we hoped to have identified the owner by doing this, in some cases we may have obtained human demographic data on another person in the household. We did find that lost neutered pets, lost pets belonging to respondents with more education, and lost cats belonging to respondents with higher income were more likely to be reunited with their owners. This could be due to different behaviors of neutered pets or to different behaviors by owners of neutered pets. Since households with higher owner education and income levels were more likely to have neutered pets, these results could be due to some complex inter-relationships, which we were not able to study further due to our limited samples size. This does suggest future avenues for investigation evaluating other pet-keeping and health variables and their associations with human lifestyle and demographic variables.

With a similar percentage of cats and dogs lost, the lower use of ID and low return due to ID in cats compared to dogs, suggests that additional efforts are needed to reunite cats and their owners. In the present study, ID tags were responsible for 15% (9–23%) of dogs getting home; fewer cats were wearing ID and only one cat owner (0.04–10%) reported that the tag was the primary way the cat was returned home. Two thirds of the lost cats who were not found did not have any identification, making it very difficult if people found these cats to locate the owners. Recent studies on ID tag use found that the most common reason for not tagging cats was because the cat was indoor-only followed by the cat was uncomfortable wearing a collar [10,11]. Refuting this justification, indoor-only cats do get lost. In one localized study, 41% of owners who were searching for their lost cat reported the cat was indoor-only [6]. Since recent studies show that the majority of cats that have a collar and tag placed on them will wear them, this could be a method to improve reunion of cats and their owners [10,12]. 

The search methods for finding these pets varied significantly. Dogs were most likely to be recovered by searching the neighborhood while the majority of cats were reunited with their owners (59%, 45–72%) by returning on their own. This finding is similar to findings reported by Lord *et al.* conducted in an Ohio study where 66% of the cats that were recovered by owners returned home on their own [6]. This difference in species search methods and returning home supports anecdotal information from shelters that many cat owners do not reach out to the shelter until the cat has been lost for several days. Lord’s research [6,7] found cat owners tended to wait three days before searching for their cats compared to one day for dogs. Furthermore, cat owners waited eight days between visits to the shelter for cats *vs*. three days for dog owners. 

Of the 110 dogs and 74 cats that had been reported as becoming lost, only nine (seven dogs and two cats) were found at animal control or through law enforcement. Of the cat owners who did not recover their cats (18 out of 74), only four searched at the shelter. Since the majority of cat owners that lose and do not recover their cat did not search at the animal shelter, there is likely an opportunity to increase messaging regarding this option as a search method for cat owners. 

Using these data to make some estimates regarding the numbers of lost pets in the US, we extrapolated based on the APPA 201 pet estimates [1]. Using the percentage of dogs that were lost in five years from our data set, of the 10,948,000 dogs that would have been lost nationally in the past five years, 10,181,640 were reunited with their owners and 766,360 were not. For cats, the numbers are even more compelling, with an estimated 12,960,000 cats becoming lost in five years, 3,240,000 estimated not recovered. These results may suggest that a proportion of “stray” pets in shelters are actually lost as opposed to abandoned by their owners. Lost pets in shelters may not be reunited with their owners if the owners do not know that there is a shelter that might have their pets.

Animal shelter staff and veterinarians can provide a valuable service by making available information on how owners of lost pets can best find their pets. They could also help owners find their pets by instituting matching of reported lost pet records with reported found pet records. Veterinarians might offer microchip and identification tag clinics for community pet owners and be sure their own clientele’s pets have microchips, collars and personalized identification. Veterinary clinics and animal shelters could have a list of resources and options for advertising for lost pets; some local papers will publish a lost pet ad free and many shelters have lost and found sections. Local veterinary associations could support advertisements for lost pets. 

Our distribution of respondents by state was similar to the US pet owning population [9]. However, we found a somewhat lower pet ownership rate than has been reported [1]. This could have been due to the RDD methodology we used, since other national estimates used household panels (pre-recruited consumers who agree to participate in future surveys), which may result in different population samples or biases [1,9]. Random digit dialing telephone surveys can miss households without a land-line. The extent and direction of this bias is unknown. We were unable to obtain the desired sample size of 400 lost pets because the actual frequency of pet loss was lower than anticipated.

Social desirability bias (causing a respondent to answer in what is perceived to be the societal norm) [13] might have led to an increase in reporting of ID on pets. In addition, this bias can lead to missing data on questions such as income and owner education as was seen here and which was anticipated. We had hoped that the telephone interview using experienced interviewers would minimize this problem. Finally, we chose to include a maximum of one dog and one cat in each household to be able to consider each pet as independent and to decrease interview time. It is possible, although no data exist to support this, that pets living in the same household might be treated more similarly than pets living in different households with regard to ID and search methods. While there were some households that lost both a dog and a cat, the difference in perceptions, values and pet keeping relating to these two different species likely decreased the dependence of this response [11].

Overall, our results indicate that about 14% of dogs and 15% of cats owned in the US became lost at least once during a five-year period. Seven percent of dogs and 25% of cats were never reunited with their owners. These data are the first to provide pet owners, veterinarians and animal welfare professionals with factual information about the frequency of pets becoming lost from the home. Among pets that were reunited with their owners, dogs were found most commonly by actively searching the neighborhood while cats most commonly came home on their own. Since 25% of lost cats were not found, other methods of reuniting cats and their owners are needed. It is possible that collars and ID tags or humane trapping could be valuable and more work is needed to determine this. Veterinarians and animal welfare professional can play a key role in helping pet owners if their dogs or cats become lost by guiding owners to use active methods to find lost pets, particularly within the owners’ neighborhoods. 

## 5. Conclusions

Overall, our results indicate that about 14% of dogs and 15% of cats owned in the US became lost at least once during a five-year period. Seven percent of dogs and 25% of cats were never reunited with their owners. These data are the first to provide pet owners, veterinarians and animal welfare professionals with factual information about the frequency of pets becoming lost from the home. Among pets that were reunited with their owners, dogs were found most commonly by actively searching the neighborhood while cats most commonly came home on their own. Since 25% of lost cats were not found, other methods of reuniting cats and their owners are needed. It is possible that collars and ID tags or humane trapping could be valuable and more work is needed to determine this. Veterinarians and animal welfare professional can play a key role in helping pet owners if their dogs or cats become lost by guiding owners to use active methods to find lost pets, particularly within the owners’ neighborhoods. 
